# Early Recovery of Physical Function After Total Hip Arthroplasty in a Patient With Osteopetrosis: A Case Report

**DOI:** 10.7759/cureus.52293

**Published:** 2024-01-15

**Authors:** Shusuke Nojiri, Azusa Kayamoto, Chiaki Terai, Yusuke Osawa, Yasuhiko Takegami

**Affiliations:** 1 Department of Rehabilitation, Nagoya University Hospital, Nagoya, JPN; 2 Department of Orthopaedic Surgery, Nagoya University Graduate School of Medicine, Nagoya, JPN

**Keywords:** osteopetrosis, marble bone disease, total hip arthroplasty, rehabilitation, physical function, hip abductor muscle strength, walking speed

## Abstract

Osteopetrosis is an uncommon and inherited disorder. Some disease-specific characteristics, such as diffuse osteosclerosis and a high incidence of fractures, may potentially affect postoperative rehabilitation. This report presents a case of successful rehabilitation early after total hip arthroplasty for osteopetrosis. A 56-year-old Japanese man, who was diagnosed with osteopetrosis at the age of 11, underwent total hip arthroplasty in the right hip. Full weight-bearing was allowed on the day after the operation; the postoperative rehabilitation program was proceeded based on a standard program as done after total hip arthroplasty for osteoarthritis. A shoe lift in the left leg was used in supervised walking training to correct the imbalanced alignment due to leg length discrepancy. The patient could walk independently with a cane 17 days after the operation. Three weeks after the operation, the patient demonstrated comfortable and maximal walking speed of 1.11 and 1.34 m/s, respectively, and maximal hip abductor muscle strength of 3.96 kgf･m, both of which were better than those before the operation. There were no adverse events during the postoperative rehabilitation course. These findings suggest the safety and efficacy of standard rehabilitation programs after total hip arthroplasty even in individuals with osteopetrosis. In addition, it may be important to consider the whole-body condition in the rehabilitation of individuals with osteopetrosis.

## Introduction

Osteopetrosis, also referred to as "marble bone disease," is an uncommon and inherited disorder, characterized by impaired osteoclast function resulting in hard and brittle bone. Osteopetrosis is associated with a high risk of fracture, osteoarthritis (OA), and poor fracture healing with non-union [1].

For individuals with hip OA, total hip arthroplasty (THA) is an effective treatment for reducing pain and improving joint function. Recovery of physical function, such as lower-limb muscle strength and walking speed, after THA in patients with OA is well documented [2-4]. However, in patients with osteopetrosis, THA is associated with technical difficulties and a higher risk of complications [5,6]. In addition to surgical techniques, some potential risk factors related to postoperative rehabilitation may exist in patients with osteopetrosis after THA. Patients with osteopetrosis commonly exhibit skeletal deformities, including scoliosis, spondylolisthesis, and degenerative arthritis [7]. Diffuse osteosclerosis and a high incidence of fractures are typical examples [8]. Considering the higher risk of intra- and postoperative complications, the rehabilitation course after THA in patients with osteopetrosis may differ from that in patients with OA; however, there is a lack of knowledge regarding the safety and efficacy of rehabilitation after THA in patients with osteopetrosis. This report presents a case of successful rehabilitation early after THA for osteopetrosis.

## Case presentation

Patient information

**Figure 1:**
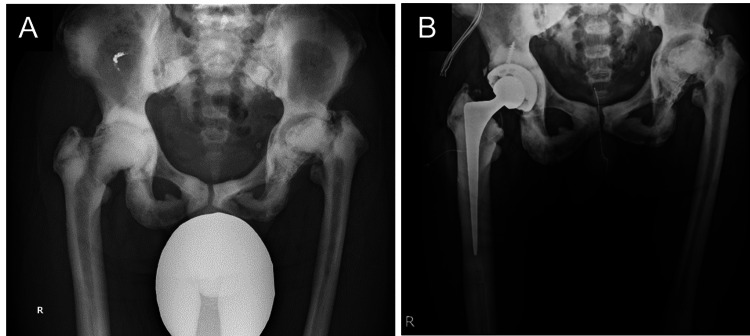
X-ray images of the hip A, preoperative X-ray image; B, postoperative X-ray image Hip osteoarthritis associated with osteopetrosis was observed in both sides.

**Figure 2:**
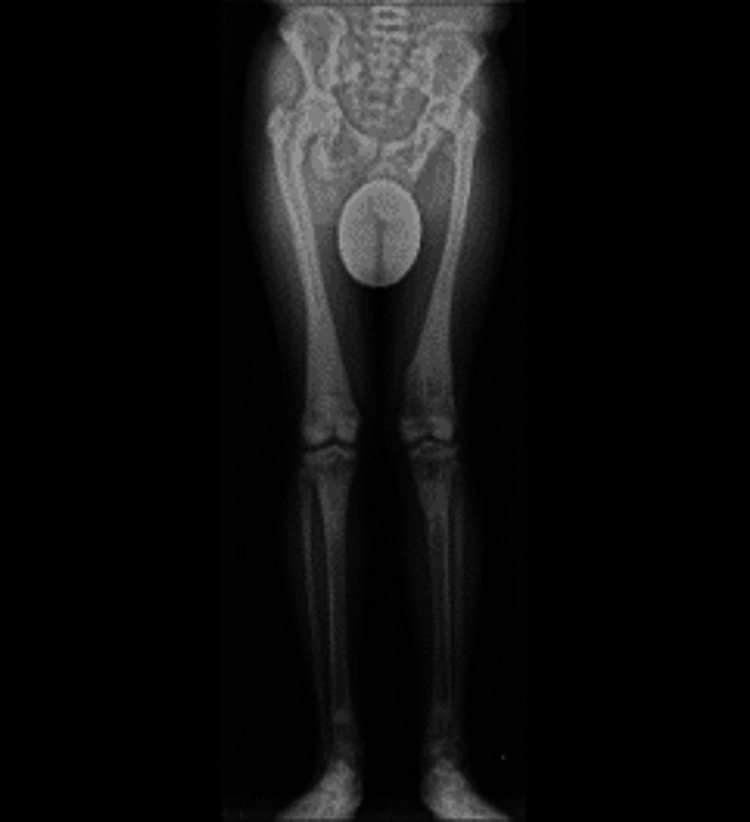
Preoperative full-leg X-ray image

**Figure 3:**
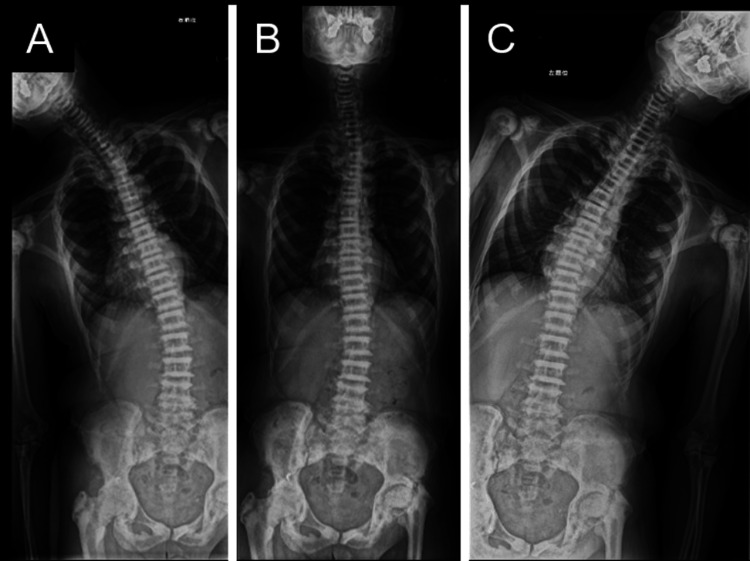
Preoperative X-ray images of the spine A, right lateral flexion; B, neutral; C, left lateral flexion Poor lateral flexion flexibility in the lumbar spine was indicated.

A 56-year-old Japanese man (height, 173 cm; body weight, 73.8 kg), who was diagnosed with osteopetrosis at the age of 11, has suffered from bilateral hip pain (left greater than right) for several decades due to hip OA associated with osteopetrosis (Figure 1A). In the last few years, however, the patient experienced rapidly worsening pain in the right hip and progressive activity limitation and therefore was scheduled for THA in the right hip. Japanese Orthopedic Association score was 51 and 61 out of 100 points, for the right and left hip, respectively [9]. Preoperative X-rays indicated leg length discrepancy (LLD) (right longer than left) due to more progressive hip OA in the left and poor lateral flexion flexibility in the lumbar spine (Figures 2, 3).

The patient had a fracture history in the rib and was treated conservatively. The patient’s father and sister also have a known history of osteopetrosis. As scheduled, THA in the right hip was performed via posterior approach without any intraoperative complications (Figure 1B). The operation time and blood loss were 142 minutes and 597 mL, respectively. There were no adverse events during the hospitalization. On postoperative day (POD) 29, the patient was transferred to a different hospital to continue rehabilitation training as the patient expected.

Preoperative physical function

Preoperative physical function was assessed on the day before the operation (Table 1). Lower limb muscle strength (hip abduction and knee extension) was measured during a 5-s isometric contraction using a handheld dynamometer with a restraining belt (μTas; Anima Corp., Tokyo, Japan). The measurement was performed in a supine with the hip and knee in a neutral position and sitting on the edge of a platform with the knee in 60-degree flexion for the hip abduction and knee extension, respectively. Comfortable and maximal walking speeds were measured over the middle 10 m of a 16-m walkway. In summary, the patient had good muscle strength in both the upper and lower limbs but demonstrated decreased walking speed (<1.0 m/s). In addition, the patient felt a perceived LLD of 1.5 cm in the right limb longer than the left.

**Table 1:** Preoperative physical function PLLD, perceived leg length discrepancy

	Preoperation
Range of motion (degree; right/left)	
Hip flexion	90/80
Hip extension	0/0
Hip abduction	-5/10
Hip adduction	15/10
Muscle strength	
Handgrip (kgf; right/left)	42.2/40.4
Hip abduction (kgf･m; right/left)	2.67/2.88
Knee extension (kgf･m; right/left)	12.15/15.26
Walking ability	
Comfortable speed (m/s)	0.76
Maximal speed (m/s)	0.90
Walking aid	none
PLLD (cm)	1.5 (right longer than left)

Postoperative rehabilitation course

**Figure 4:**
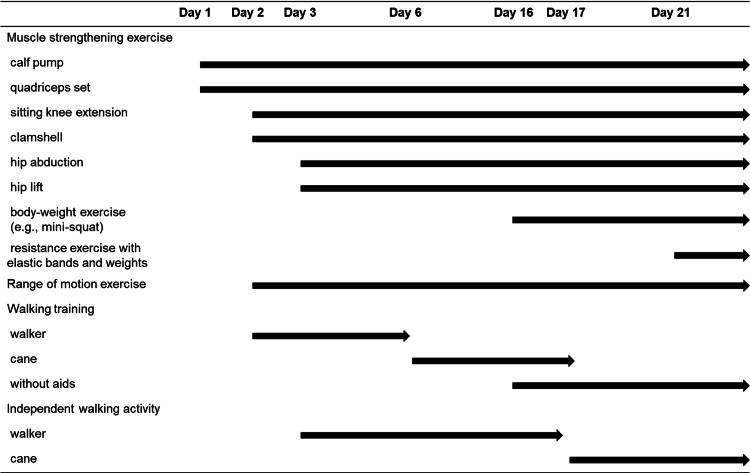
Postoperative rehabilitation course

The postoperative rehabilitation course is presented in Figure 4. Full weight-bearing was allowed from POD 1. On POD 1, the patient was able to stand up and transfer to the wheelchair. The patient started supervised walking training on POD 2 using walking aids and a shoe lift in the left leg for correcting LLD. Shoe lift, started from 4.0 cm on POD 2, remained in use at the supervised training and was decreased gradually. On POD 3, the patient achieved unsupervised walking with a walker; however, the patient was instructed not to increase physical activity because of the imbalanced alignment due to the LLD. On POD 9, the appropriate shoe lift was decided as 2.5 cm based on the structural LLD and prescribed. On POD 17, the patient received the lifted shoe; at the time, the patient was allowed to walk independently with a cane and instructed to increase physical activity. Enhanced muscle strengthening exercise using elastic bands and weights was added on POD 21.

Postoperative physical function

Postoperative physical function was assessed via lower-limb muscle strength and walking speed on POD 20 and POD 27 (Table 2). Except for the knee extension muscle strength, the patient demonstrated better performance than preoperation at both postoperative time points. Although the knee extension muscle strength decreased at POD 20, it recovered to almost the same extent as preoperative at POD 27. The perceived LLD remained at 4.0 cm on POD 27.

**Table 2:** Change in physical function NRS, numerical rating scale; PLLD, perceived leg length discrepancy; POD, postoperative day

	Preoperation	POD 20	POD 27	
Muscle strength in the right lower limb (kgf･m)				
Hip abduction	2.67	3.96	4.50	
Knee extension	12.15	7.38	11.50	
Walking ability				
Comfortable speed (m/s)	0.76	1.11	0.99	
Maximal speed (m/s)	0.90	1.34	1.39	
Walking aid	None			
Hip pain (NRS)	4	0	0	
PLLD (cm; right longer than left)	1.5	-	4.0	

## Discussion

In this report, we described a rehabilitation course and functional recovery after THA in a patient with osteopetrosis. Although slight modifications by the shoe list for correcting LLD were added, we basically provided a standard rehabilitation program after THA, as is done in patients with OA (e.g., gradually increasing muscle strength exercise and walking training). Our result suggests the safety of a standard rehabilitation program after THA even in patients with osteopetrosis.

In individuals with osteopetrosis, some disease-specific factors, such as diffuse osteosclerosis and high incidence of fractures, could have a negative impact on postoperative rehabilitation. In the present case, there were no adverse events during the postoperative rehabilitation course, both in affected and non-affected regions. These results imply the safety of a standard rehabilitation and reinforce the suggestion by Gao et al. [10]. They reported a case of a 52-year-old female patient with osteopetrosis who was able to walk with sticks 18 days after THA and stated that postoperative functional exercises for patients with osteopetrosis ought to be carried out “as usual.”

Our interventions of taking into consideration the whole body, such as the contralateral hip and spine, may have contributed to the safe and effective rehabilitation course. In individuals with osteopetrosis, in particular, it may be important for therapists to consider the whole-body condition, since osteopetrosis often presents non-localized problems. Difficulties in surgical techniques may also be related to this perspective; sometimes, LLD could not be able to corrected with the contralateral THA. In this case, the patient had other problems than hip OA in the right hip, such as more progressive hip OA in the left and poor spine lateral flexion flexibility. Hence, to avoid the overloads in the abovementioned regions, we used a shoe lift in the contralateral leg to correct LLD in the early phase after THA, and we instructed the patient not to increase physical activity until the shoe lift was ready.

In terms of functional recovery, such as muscle strength and walking function, the early recovery in the present case is comparable or somewhat superior to that after THA in patients with OA. Previous studies regarding functional recovery after THA in patients with OA have reported that physical function decreased one month after the operation compared to preoperation and recovered later [3,4]. In this case, better hip abduction muscle strength and walking speed than preoperation were observed three weeks after the operation. When the postoperative rehabilitation is carried out safely, good recovery in muscle strength and walking function could be expected even in individuals with osteopetrosis. Another potential reason for the early recovery in this case is good preoperative physical function. It has been well established that preoperative physical function, including nonoperating regions, is an important factor for postoperative rehabilitation courses and clinical outcomes [11-13]. The patient in the present case had a relatively good preoperative physical performance compared to those undergoing THA, and therefore the early recovery was achieved.

## Conclusions

In this case report, we describe a rehabilitation course and functional recovery after THA in a patient with osteopetrosis. Our intervention with the consideration for the whole-body condition may have contributed to the safety rehabilitation. The patient showed good recovery of muscle strength and walking speed within one month. Even in individuals with osteopetrosis, good recovery in muscle function may be expected, as a result of safety rehabilitation. In rehabilitation of patients with osteopetrosis, skeletal complications, such as fractures, should be noted. Further studies with larger sample sizes would be needed to confirm our findings.
